# Detection and Characterisation of Conductive Objects Using Electromagnetic Induction and a Fluxgate Magnetometer

**DOI:** 10.3390/s22165934

**Published:** 2022-08-09

**Authors:** Lucy Elson, Adil Meraki, Lucas M. Rushton, Tadas Pyragius, Kasper Jensen

**Affiliations:** 1 School of Physics and Astronomy, University of Nottingham, University Park, Nottingham NG7 2RD, UK; 2 Tokamak Energy, 173 Brook Dr, Milton, Abingdon OX14 4SD, UK

**Keywords:** electromagnetic induction, conductivity, eddy current, magnetic field, non-destructive testing, numerical simulation, magnetic permeability, fluxgate magnetometer

## Abstract

Eddy currents induced in electrically conductive objects can be used to locate metallic objects as well as to assess the properties of materials non-destructively without physical contact. This technique is useful for material identification, such as measuring conductivity and for discriminating whether a sample is magnetic or non-magnetic. In this study, we carried out experiments and numerical simulations for the evaluation of conductive objects. We investigated the frequency dependence of the secondary magnetic field generated by induced eddy currents when a conductive object is placed in a primary oscillating magnetic field. According to electromagnetic theory, conductive objects have different responses at different frequencies. Using a table-top setup consisting of a fluxgate magnetometer and a primary coil generating a magnetic field with frequency up to 1 kHz, we were able to detect aluminium and steel cylinders using the principle of electromagnetic induction. The experimental results were compared to numerical simulations, with good overall agreement. This technique enables the identification and characterisation of objects using their electrical conductivity and magnetic permeability.

## 1. Introduction

Electromagnetic induction is routinely used in eddy current testing as a non-destructive technique for flaw detection and material characterisation [1,2,3,4]. This technique offers the advantage of non-contact scanning without causing damage to the sample under test. Such measurements have various applications, for example, in the detection, localisation, and characterisation of metallic objects in the defence, aerospace, and quality control industries [5,6,7,8,9]. Flaw detection through the use of eddy current induction has already shown potential in a number of industries, including aerospace, transport, and power supply [10]. Applications within aerospace and related industries include surface and subsurface crack detection and subsurface corrosion detection [11]. Additional applications include weld inspection, tube inspection, and material verification. Current technology typically uses frequencies on the order of kHz to MHz, which confer the potential to detect cracks ∼0.1 mm or less in depth [1,10]. The method is based on detecting and characterising electrically conductive objects using an active excitation, wherein an oscillating primary magnetic field $B_{1}\left(t\right)$ created by a coil induces eddy currents in the object. The eddy currents then create a secondary magnetic field, $B_{\mathrm{ec}}\left(t\right)$, which can be measured by a sensitive magnetometer such as a fluxgate magnetometer. This technique can be used to detect a wide range of objects, as it is sensitive to both the electrical conductivity σ and the magnetic permeability $\mu =\mu_{0}\mu_{r}$ of the object, where $\mu_{0}$ is the vacuum permeability and $\mu_{r}$ is the relative permeability. It has been shown that measuring both the amplitude and phase of the magnetic field can be used to reconstruct the eddy currents. This principle finds applications in various areas, such as in the monitoring of fuel cells [12,13].

A major challenge when detecting a metallic object is discriminating the object, such as an unexploded ordnance (UXO), from the noisy environment it is in [14]. It takes time and resources to identify the object, especially due to false signals from other metal objects and cultural features such as metal buildings, pipelines, and oil well casings. By measuring the secondary magnetic field of an electrically conductive object which is placed in a low frequency primary magnetic field, distinct spectral characteristics such as electrical conductivity, magnetic permeability, object geometry, and size can be obtained [15,16,17].

In this work, we have built a tabletop setup with coils and a commercial fluxgate magnetometer. With this setup, we carried out a systematic study in which a number of metallic objects were detected at different positions and with different excitation frequencies. From the frequency dependence of the measured induced magnetic field, we extracted values for the electrical conductivity and magnetic permeability of the objects by fitting experimental data to analytical formulae. In order to validate our experimental results, we built a range of different COMSOL models and made comparisons between the experimental results and numerical simulations. The results are in good agreement with the numerical simulations performed in COMSOL.

The rest of this work is organised as follows: first, we describe the experimental setup, and methods, and different configurations in which the metallic objects were placed, including on-axis eddy current measurements for (a) varying the frequency of the primary oscillating magnetic field and (b) varying the position of the object along the *z*-axis relative to the excitation coil and magnetometer. We then present off-axis measurements, in which the primary coil and magnetometer are fixed in position while the object is moved off-axis (i.e., along the *y*-axis at a fixed *z*-position). We present results for solid and hollow cylinders made of aluminium and steel.

## 2. Setup and Methods

**Figure 1:**
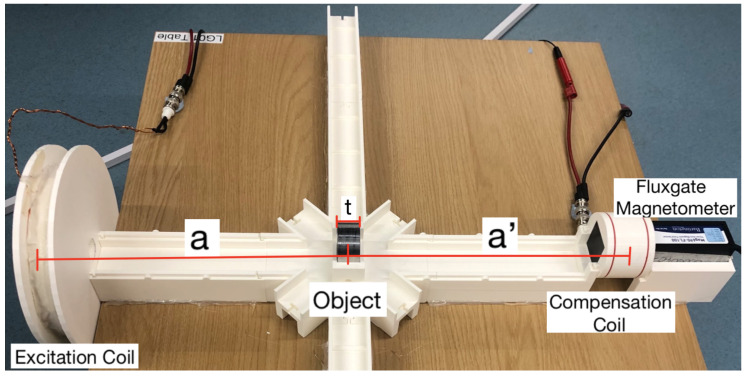
Tabletop active detection system, consisting of an excitation coil, a fluxgate magnetometer, a compensation coil, and an object, of thickness t, which can be placed either on-axis or off-axis. The object is positioned at a distance a from the excitation coil and a distance a’ from the detection point of the sensor.

Our tabletop setup for detecting and characterising metallic samples is shown in Figure 1. The setup is 3D printed, which allows the components to be placed with high precision. The experiments were controlled with an sbRIO-9627 field-programmable gate array (FPGA) programmed in LabVIEW. The FPGA can output sinusoidal signals, record data, perform lock-in amplification, apply real-time feedback, and analyse data. Magnetic fields are detected with a Bartington MAG690 fluxgate magnetometer, which has a scale factor of 100 mV/μT and a bandwidth of ≈1 kHz. The fluxgate magnetometer measures all three components of the magnetic field, although for simplicity only the *z*-component was recorded. In our experiments, the data were obtained with the magnetic field oscillating at a particular frequency ν. The resulting oscillating signal, $S\left(t\right)=R\cos\left(2\pi \nu t+\phi \right)=I\cos\left(2\pi \nu t\right)-Q\sin\left(2\pi \nu t\right)$, can be decomposed into the in-phase *I* and out-of-phase *Q* components. These components are detected with a lock-in amplifier implemented via the FPGA.

Two coils, an excitation coil and a compensation coil, are used for generating magnetic fields. The excitation coil produces a primary field $B_{1}\left(t\right)$ oscillating at a particular frequency ranging between 10–1000 Hz. The reference phase of the lock-in amplifier was adjusted such that the primary field was detected in the in-phase component *I* only. A compensation coil with a one-turn Helmholtz configuration and a radius of 3 cm was used, placed around the magnetometer’s detection point. We placed a 6 Ω power resistor in series with the compensation coil. This creates an additional magnetic field $B_{2}\left(t\right)$, the ‘compensation field’, at the same frequency as the primary field, cancelling the primary magnetic field at the position of the magnetometer such that the total magnetic field $B_{1}\left(t\right)+B_{2}\left(t\right)\approx 0$ in the absence of an object.

The excitation coil has an 8 cm radius, 60 windings, and is positioned such that the centre of the coil is 48.4 cm away from the detection point of the magnetometer. We used a relatively thick wire with a diameter of 1 mm, which leads to a small resistance and negligible heating in our excitation coil. Furthermore, a 10 Ω power resistor was placed in series. In order to produce a magnetic field, a sinusoidal voltage of ≈7.2 V (peak to peak) is sent to the excitation coil and the phase is adjusted such that the signal is in the in-phase component of the lock-in output. The applied voltage generates a current of $I_{e}=0.53$ A in the excitation coil corresponding to a magnetic dipole moment of $\mu_{e}=0.64$ Am${}^{2}$ (pointing in the *z*-direction). The magnetic field produced is $B_{1}=1.09\;\mu$T at the position of the magnetometer. To compensate for this field, a 0.24 V (peak to peak) oscillating voltage is applied to the compensation coil. This generates a current of $I_{c}=0.036$ A in the compensation coil corresponding to a magnetic dipole moment of $\mu_{c}=1.02\times 10^{-4}$ Am${}^{2}$.

When a conductive object is placed between the compensation coil and the excitation coil, eddy currents are induced in the object, producing a secondary magnetic field (or an ‘induced field’) $B_{\mathrm{ec}}\left(t\right)$ oscillating at the same frequency as the primary field. Note that the eddy currents are mainly generated by the primary field, as the compensation field is small (compared to the primary field) at the position of the object. The amplitude of the secondary field is therefore proportional to the amplitude of the primary field $|B_{\mathrm{ec}}\left|\propto \right|B_{1}|$. Due to the applied compensation field, the magnetometer directly measures the secondary field as the total oscillating field $B_{\mathrm{tot}}\left(t\right)=B_{1}\left(t\right)+B_{2}\left(t\right)+B_{\mathrm{ec}}\left(t\right)\approx B_{\mathrm{ec}}\left(t\right)$ at the magnetometer’s position. Applying a compensation field in order to measure the secondary field directly can be convenient; when using an optically pumped magnetometer for detecting the magnetic field, the signal-to-noise ratio of the measurement can improve by several orders of magnitude [18,19,20]. However, we note that the stability and noise in our measurements with the fluxgate magnetometer was independent of whether the compensation field was applied or not (see Appendix B).

**Figure 2:**
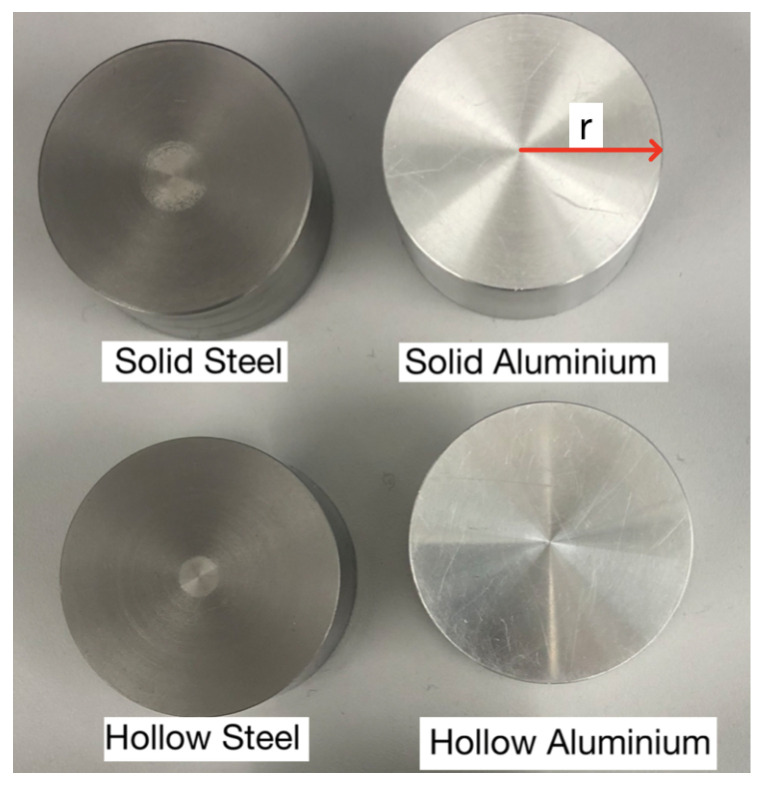
6061 T6 aluminium and 440c steel samples.

We detected and characterised four different samples (see Figure 2). The samples were solid/hollow cylinders with radii of 2 cm and width 2 cm. The hollow cylinders had a thickness of 4 mm. The cylinders were made of either 6061 T6 aluminium or 440c steel; 6061 T6 aluminium has an electrical conductivity of σ=24.6 MS/m, is non-magnetic, and has a relative magnetic permeability of $\mu_{r}=1$, while 440c steel has an unknown relative permeability. While the electrical conductivity is unknown, it can be determined experimentally [21]. We chose to study aluminium and steel samples in order to highlight the differences between non-magnetic and magnetically permeable materials. Other metals, such as brass, copper, and ferrite, can be studied and differentiated using electromagnetic induction [22].

Using our tabletop setup, we measured how the secondary field depends on the frequency of the primary field and on the distance from the excitation coil to the sample, and hence from the sample to the fluxgate. For on-axis measurements, the object was placed directly between the excitation coil and the magnetometer. In order to study how variations in the frequency affect the induced eddy currents, the sample was placed 22.4 cm away from the front of the excitation coil and the frequency was varied between 10 Hz and 1 kHz. When varying the distance of the object, a constant frequency of 500 Hz was used. The conductive objects were placed at approximately 5 cm intervals, beginning at 5 cm from the front of the excitation coil to 39.5 cm away. The off-axis measurements were carried out with the samples approximately half-way between the two coils, that is, 22.4 cm away from the front of the excitation coil. The conductive objects were placed from 0 cm to 34 cm off-axis, then the induced magnetic field was measured.

## 3. Numerical Simulations

**Figure 3:**
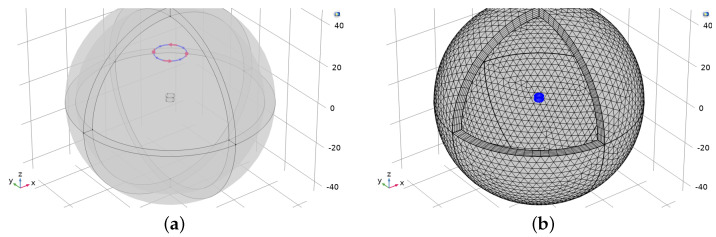
Diagrams of the 3D finite element model (COMSOL) showing (**a**) an object at the origin, the primary coil, and the boundary layer; and (**b**) the free tetrahedral elements for the object and for the finite domain employed in this study.

Eddy current simulations were performed in COMSOL Multiphysics 5.6 using the AC/DC module. The Magnetic Fields interface was used to compute the magnetic field and induced current distributions in and around the coils and conductors. The default settings in COMSOL were used for this interface. The experimental setup was built as a 3D model (Figure 3). The model consisted of a circular coil placed above a metallic object. To reduce complexity, an imaginary single-turn coil was chosen for the primary magnetic field. The coil and the object were placed in the finite sphere air domain, the size of which was ten times bigger than the size of the object. As seen in Figure 3, the model includes the infinite element domain, which is one-tenth of the overall dimension of the model. The functionality of the infinite domain means that the governing equations behave similarly to nature and achieve a non-reflecting boundary condition. The finite element mesh was used to subdivide the CAD model into smaller domains, where a set of equations can be solved. As these elements are made as small as possible (i.e., the mesh is refined), the solution approaches the true solution. We performed subsequent mesh refinements until no appreciable changes occurred in the simulated curves; these results are shown in Appendix A. Figure 3b shows that the finite element mesh consists of three-dimensional tetrahedral solid elements and five layers of infinite element meshes, which have been added to the spherical domain. All of the simulations were performed on a workstation using a 3.60 GHz Intel(R) Xeon processor with 128 GB RAM.

Each simulation was run twice, first with the object present (matching the properties of those used experimentally) and then again without the object present. Instead of removing the object from the simulation, its properties (most notably its electrical conductivity and magnetic permeability) were changed to match that of the host medium (air). When using this technique, the mesh was preserved in both cases, eliminating the influence of the mesh on the results. The difference between these two simulation outputs is the magnetic field induced in the object.

**Figure 4:**
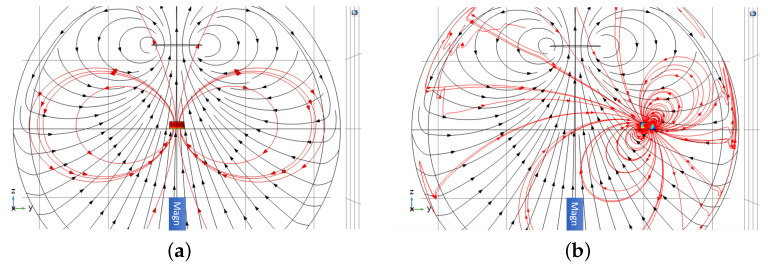
Simulation model results when a solid metallic cylinder is placed (**a**) on-axis and (**b**) off-axis. The magnetic moment of the primary coil (black line) is aligned along the *z*-axis, and stream plots of the magnetic filed lines (black lines with arrows) produced by the primary coil are shown. The induced secondary magnetic field $B_{\mathrm{ec}}\left(t\right)$ generated by eddy currents in the object are depicted by the red lines and arrows.

Figure 4 shows the directions of the primary and secondary magnetic fields when an object is placed on-axis and off-axis, respectively. When the object is placed on-axis, the primary and secondary magnetic fields only have a *z*-component at the magnetometer position. When the object is placed off-axis in the *y*-*z*-plane, the secondary field generally has both *y*- and *z*-components. In the following section, we present experimentally measured values for the *z*-component of the secondary field and compare them to the values from the numerical simulations.

## 4. Results and Discussion

**Figure 5:**
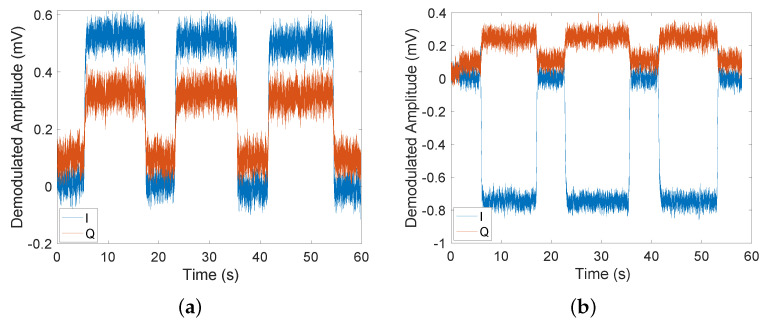
Time traces of the lock-in outputs when detecting conductive objects with the fluxgate magnetometer and the active detection setup for (**a**) the solid aluminium cylinder and (**b**) the solid steel cylinder. The conductive object was placed 22.4 cm away from the excitation coil. For these measurements, the primary magnetic field was 1.09μT at the magnetometer position and oscillated at a frequency of 500 Hz.

**Figure 6:**
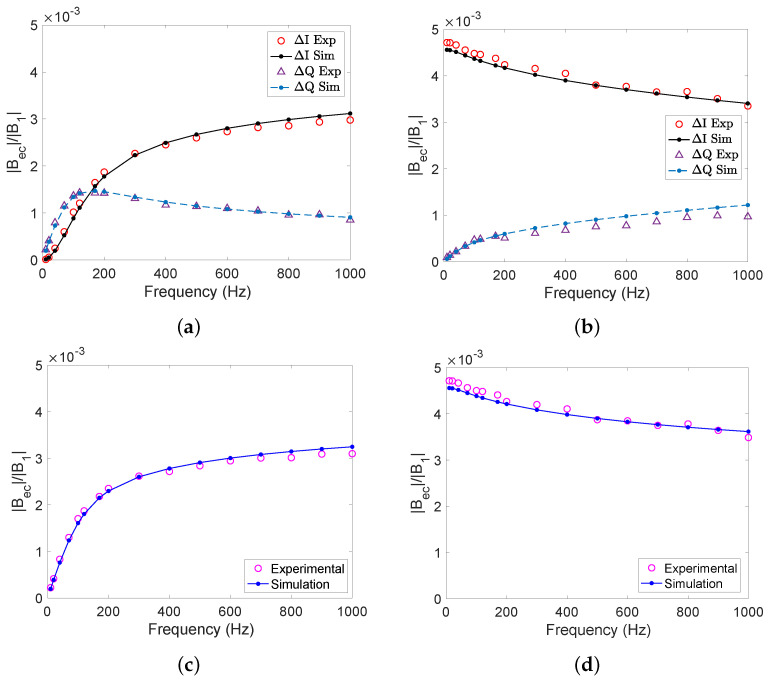

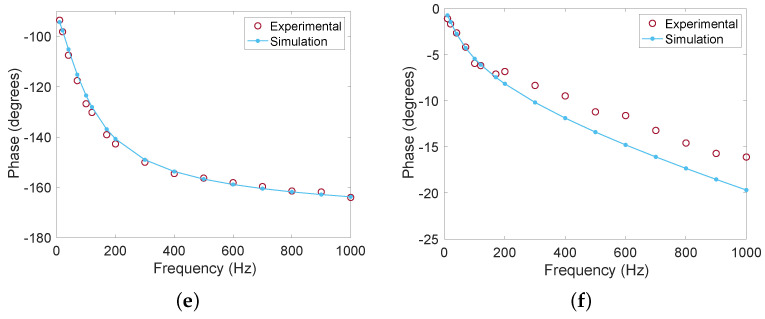
Experimental and simulation results for the secondary magnetic field for solid aluminium, (**a**,**c**,**e**), and steel cylinders, (**b**,**d**,**f**), when varying the frequency between 10 Hz to 1 kHz. (**a**,**b**): In-phase *I* and out-of-phase *Q* components; (**c**,**d**): ratio of the amplitude of the secondary magnetic field to the primary magnetic field at the magnetometer position (**e**,**f**): phase (in degrees) of the secondary magnetic field with respect to the primary magnetic field.

Figure 5 shows examples of experimentally recorded time traces when conductive objects were placed into the setup for ∼10 s and then taken back out for ∼5 s. Three repeats of this measurement were taken. The time traces show the demodulated fluxgate magnetometer signals *I* and *Q* for an excitation frequency of 500 Hz when aluminium and steel cylinders, respectively, were placed into the setup. Using these time traces, the in-phase ΔI and out-of-phase ΔQ components of the secondary magnetic field can be found by subtracting the signals with and without the object. The standard deviation (SD) of ΔI and ΔQ was ∼0.03 mV in all time traces for both the aluminium and steel samples. We found that the in-phase component for aluminium (Figure 5a) has a signal-to-noise ratio (SNR) of ∼17, while the out-of-phase component has an SNR of ∼7. Similarly, we can see that for steel (Figure 5b) the SNR is ∼24 for the in-phase component of the field and ∼4 for the out-of-phase component. The values for ΔI and ΔQ can then be used to calculate the magnitude of the secondary field relative to the primary magnetic field $|B_{\mathrm{ec}}\left|/\right|B_{1}|$ measured at the magnetometer position as well as the phase ϕ of the secondary magnetic field with respect to the primary magnetic field. In oder to account for the bandwidth of the magnetometer, the primary field was recorded at every frequency. Time traces were taken for a range of frequencies between 10 Hz and 1 kHz; the results are shown in Figure 6.

### 4.1. Varying Frequency

Figure 6 shows the detected secondary magnetic field for solid aluminium (Figure 6a,c,e) and steel (Figure 6b,d,f) cylinders as functions of the excitation frequency, *f*. The figure shows the in-phase ΔI and out-of phase ΔQ components of the secondary field normalised to the amplitude of the primary field (Figure 6a,b), the normalised amplitude of the secondary field (Figure 6c,d), and the phase $\phi =\tan^{-1}\left(\Delta Q/\Delta I\right)$ of the secondary field relative to the primary field (Figure 6c,d).

For the aluminium sample, the out-of-phase component ΔQ is linear up to around 50 Hz and dominates (i.e., is larger than ΔI) up to 150 Hz (see Figure 6a). The overall magnetic field ratio saturates at ∼350 Hz (see Figure 6c) due to the skin effect. The skin effect becomes important when the skin depth $\delta =1/\sqrt{\pi \mu \sigma f}$ becomes comparable to or smaller than the thickness *t* of the object, corresponding to frequencies $f\geq 1/\left(t^{2}\pi \mu \sigma \right)=26$ Hz when using *t* = 2 cm, $\mu =\mu_{0}$ and σ = 24.6 MS/m for our aluminium sample. For a non-magnetic sample, such as our aluminium sample, the phase $\left|\phi \right|∼90^{\circ }$ at low frequencies, when the signal is mainly in the out-of-phase component, and approaches $\left|\phi \right|∼180^{\circ }$ at higher frequencies, when the in-phase component dominates (see Figure 6e). This is due to the secondary field being in the opposite direction to the primary field at high frequencies. The conductivity of our aluminium sample can be extracted from the gradient $\left(|B_{\mathrm{ec}}\left|/\right|B_{1}|\right)/f$ at low frequencies. This is achieved by comparing the experimentally determined gradient to an analytical formula valid for a non-magnetic conductive cylinder. Detailed calculations based on [23] are presented in Appendix C. Using the gradient of the magnetic field ratio up to 20 Hz we determined the conductivity to be 25.5(±1.8) MS/m, which is in agreement with the expected value of 24.6 MS/m for 6061 T6 aluminium. Figure 6a,c,e show the results of the numerical simulations carried out in COMSOL, which agree very well with these experimental results.

For our steel sample, the largest signal is seen at low frequencies, as shown in Figure 6b. This is because steel is magnetically permeable. The secondary field is produced in the same direction as the primary field due to steel being ferromagnetic. As the in-phase component dominates at all frequencies, as expected the phase of the signal is small $|\phi |<20^{\circ }$ (Figure 6f) and the magnetic field ratio (Figure 6d) is very similar to that of the in-phase component. While the overall detected magnetic field decreases slightly with frequency, its signal remains a large one. It can be seen that at higher frequencies the out of-phase component increases as the in-phase component decreases.

**Figure A4:**
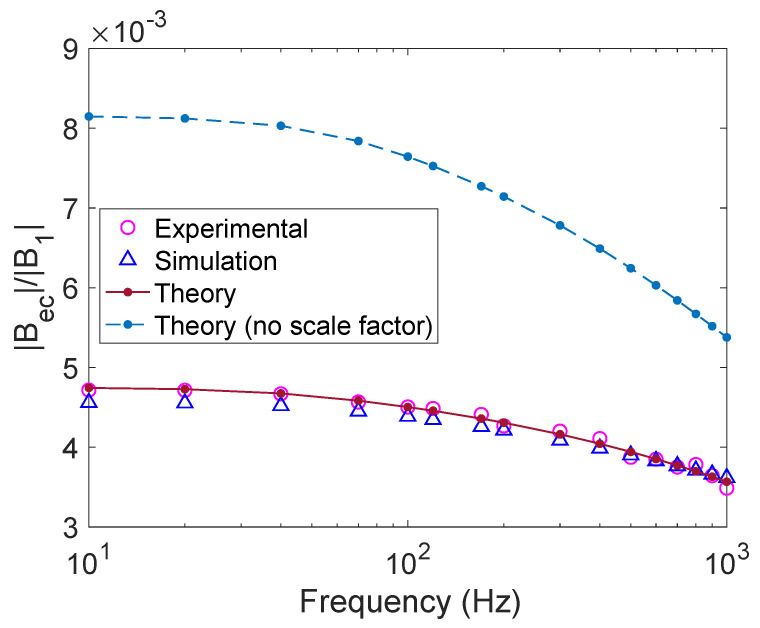
Fitting the conductivity and permeability of 440c steel using equations similar to those found in [21].

The exact value of the magnetic permeability of the 440c steel samples we used was not known to us in advance. In [21], their 440c steel samples were found to have a value of $\mu_{r}=$ 16–17. However, no other studies could be found in which the magnetic permeability of 440c steel has been calculated, and thus it is unknown how much this value changes between samples. For low permeabilities, a small change in permeability can cause a large change in the detected signal [21,24]. As $\mu_{r}\gg 1$, the change in the signal is much smaller. Hence, in order for a simulation comparison to be performed, the conductivity and permeability first need to be determined experimentally. To determine these values, we fitted our experimental results to analytical formulae from [21]. As those formulae are valid for a sphere in a uniform field, and our experimental objects were cylinder and were not in a uniform RF field, we included a scale factor in the fit function (see Appendix C.2). For our 440c steel sample, a permeability of $\mu_{r}=50\;\left(\pm 15\right)$ and conductivity of σ=1.67(±0.2) MS/m were obtained from the fit (see Figure A4); these values were then used in the simulations. The simulation results for the magnetic field ratio agree within ∼5% with the experimental data for these parameters, with both following the same trends. Hence, these values were used throughout the simulations.

**Figure 7:**
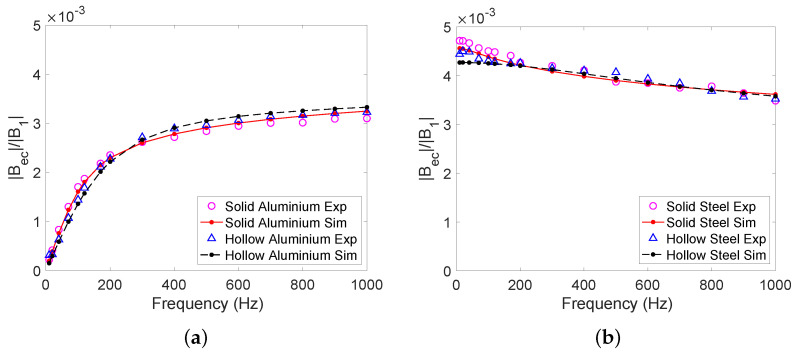
Experimental and simulated results for secondary magnetic fields from solid and hollow cylinders at frequencies between 10 Hz and 1 kHz for (**a**) 6061 T6 aluminium and (**b**) 440c steel.

The obtained results for the solid aluminium and steel cylinders are shown side-by-side in Figure 6. As can be seen, the samples can easily be differentiated by varying the excitation frequency. In particular, at low frequencies the phase of the secondary magnetic field is close to $0^{\circ }$ for steel (which is magnetic), while the phase is close to $90^{\circ }$ for aluminium (which is non-magnetic). We performed measurements with hollow aluminium and steel cylinders as well; Figure 7 shows a comparison of the magnetic field ratio $|B_{\mathrm{ec}}\left|/\right|B_{1}|$ as a function of frequency for the solid and hollow cylinders (see Figure 2). We found that the secondary field from the hollow cylinders was close to that of the solid cylinders, which is due to the objects having similar dimensions.

### 4.2. Varying Distance

**Figure 8:**
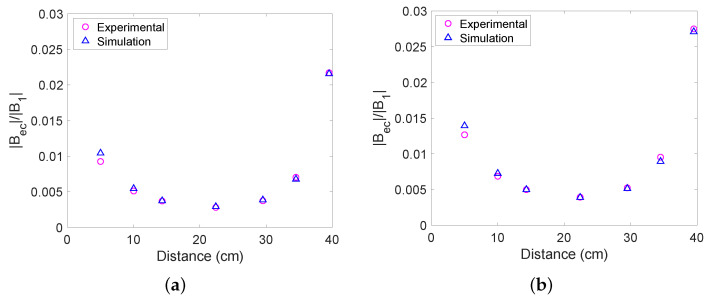
Detected magnetic field ratio as the distance from the excitation coil to the object is varied at 500 Hz for (**a**) the solid aluminium cylinder and (**b**) the solid steel cylinder.

In this subsection, we present our on-axis measurements when the objects were placed directly in between the excitation coil and the fluxgate magnetometer. The excitation coil was placed at 0 cm. The conductive objects were then placed at intervals between 5 cm and 39.5 cm away from the excitation coil. The measurements were taken at 500 Hz. This frequency was chosen based on Figure 7, as there is a large signal for both aluminium and steel. For both aluminium and steel objects, the magnetic field ratio is smallest when the object is halfway between the excitation coil and fluxgate magnetometer and largest when the object is placed near either the excitation coil or the magnetometer (see Figure 8). By comparing Figure 8a,b, it can be seen that the signal is larger for steel than aluminium, as is the case when the frequency is varied. The sample can be detected at all positions. Figure 8 shows the results of numerical simulations, again showing good agreement between experiment and simulation.

### 4.3. Off-Axis Measurements

**Figure 9:**
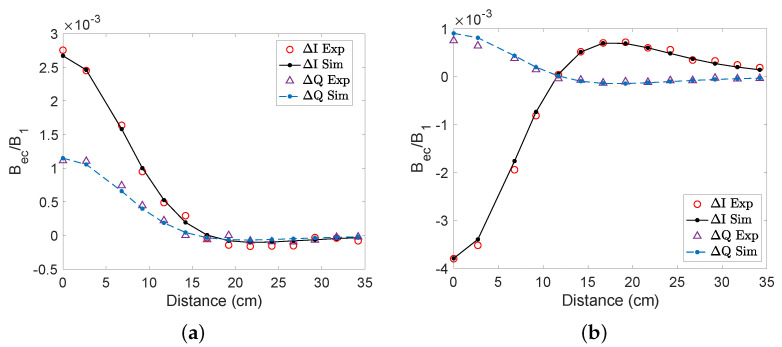
In-phase and out-of-phase components of the signal detected as the conductive objects were varied off-axis at 500 Hz for (**a**) the solid aluminium cylinder and (**b**) the solid steel cylinder.

**Figure 10:**
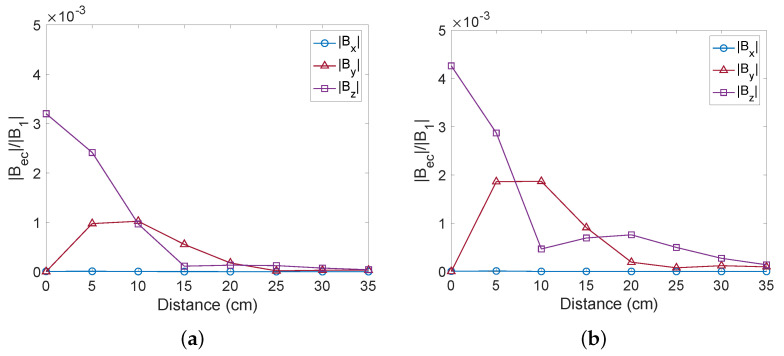
Simulated results of the induced fields in the *x*-, *y*- and *z*-direction as (**a**) the solid aluminium cylinder and (**b**) the solid steel cylinder were moved off-axis.

Here, we present our results when detecting objects placed off-axis. The object was placed at y=0 cm (on-axis) to 34.5 cm off-axis. As both the aluminium and steel cylinders are moved off-axis, the in-phase and out-of-phase components become smaller and change their signs, as shown in Figure 9. For aluminium, the signals change their sign when the object is around 16 cm off-axis. Similarly, for steel the signal changes its sign when the object is around 12 cm off-axis. These results were validated by COMSOL simulations. The reason for the change in sign is due to the orientation of the induced dipole (see Figure 4). In the experiment, only the *z*-component of the magnetic field was recorded; however, the secondary magnetic field generally has both *y*- and *z*-components when the object is placed off-axis. In order to study how the vector components of the detected secondary magnetic field change as the object is moved off the axis, we carried out COMSOL simulations in which the object was swept along the *y*-axis (see Figure 10). For the analysis of solid aluminium and steel cylinders, $B_{\mathrm{ec},x}$ and $B_{\mathrm{ec},y}$ were obtained in addition to $B_{\mathrm{ec},z}$. The signal was measured at an excitation frequency of 500 Hz in order to match the experimental conditions. We found that the *z*-component of the secondary magnetic field is maximal when the object is on-axis (at y=0), and that the *y*-component reaches a maximum at around 5 cm to 10 cm for both aluminum and steel samples. The *x*-component of the field is zero, as the induced dipole is in the *y*–*z*-plane.

## 5. Conclusions

In conclusion, we have detected and characterised non-magnetic (aluminium) and magnetic (steel) samples by inducing eddy currents in them and detecting the secondary magnetic field with a fluxgate magnetometer. We have shown that the samples can be differentiated by varying the frequency of the primary magnetic field. Their electrical conductivity and magnetic permeability were determined by fitting the experimentally measured secondary field to analytical formulae. Overall, our experimental results are in good agreement with numerical simulations carried out in COMSOL. Using a 3D printed setup was critical for achieving good agreement, as it enabled us to place samples at well-defined positions in a reproducible way. By varying the position of the sample with respect to the excitation coil and magnetometer, we were able to demonstrate the possibility of locating metallic objects based on the *x*-, *y*-, and *z*-components of the secondary magnetic field. Our experimental setup (primary coil, object, fluxgate magnetometer, and compensation coil) was placed horizontally on a table. However, by measuring all components of the magnetic field, our apparatus is able to detect objects placed below the table as well. If the same setup were modified to be used outdoors, it would thus be able to detect and characterise underground objects. Localisation of a magnetic dipole can be carried out using a small array of vector fluxgate magnetometers [25]. Using a primary field and detecting the induced secondary magnetic field has advantages in that both magnetic and non-magnetic objects can be detected. Our measurements were carried out in unshielded conditions using lock-in detection at particular discrete frequencies between 10 Hz and 1000 Hz. This method is not sensitive to the Earth’s background magnetic field (around 0.5 Gauss in our laboratory) as long as it is within the range of the magnetometer (which in this case was ±1 Gauss, although the same magnetometer is available with a ±10 Gauss range). This method can potentially be application to the detection of unexploded ordnance. For outdoor measurements, our setup could be mounted on a moving platform on land, or alternatively on an underwater glider [17] or an airborne drone [26,27]. Technical difficulties may arise due to additional noise from a moving platform. Additionally, for detection of objects at larger distances the magnetic dipole moment of the primary coil should be increased. The localisation and characterisation of samples could be further explored with the help of machine learning [28]. It is worth noting that, although we used a fluxgate magnetometer to detect eddy currents in this study, other types of sensors can be used, including optically pumped magnetometers [29,30,31,32,33] and magnetoresistive sensors [34,35]. Using a highly sensitive optically pumped magnetometer instead of a fluxgate magnetometer could potentially extend the detection range [20].

## Figures and Tables

**Figure A1:**
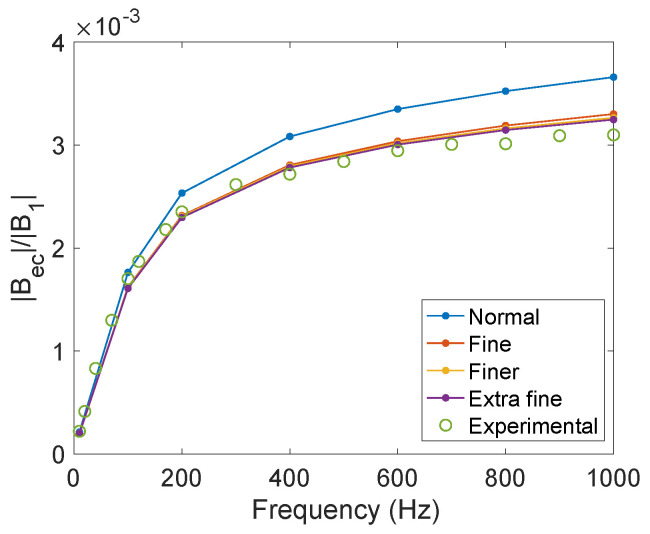
Experimental and simulated magnetic field ratio ($|B_{ec}\left|/\right|B_{1}|$) for solid aluminium cylinders with different mesh sizes: normal = 11,394; fine = 21,634; finer = 55,144; extra-fine = 188,265.

**Figure A2:**
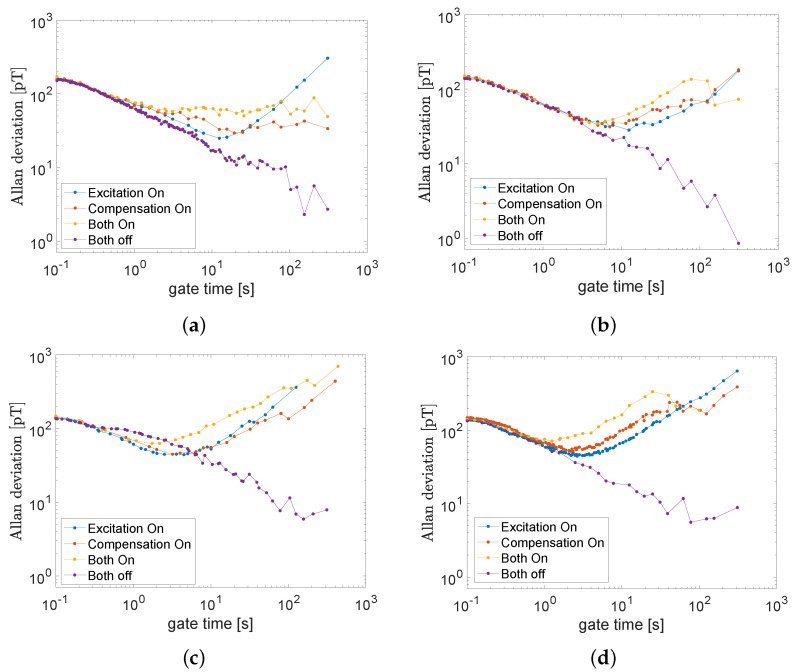
Allan deviation of 10 minute (unshielded) noise measurements with (i) excitation coil on, (ii) compensation coil on, (iii) both coils on, and (iv) no coils on at (**a**) 10 Hz, (**b**) 120 Hz, (**c**) 500 Hz, and (**d**) 1000 Hz.

**Figure A3:**
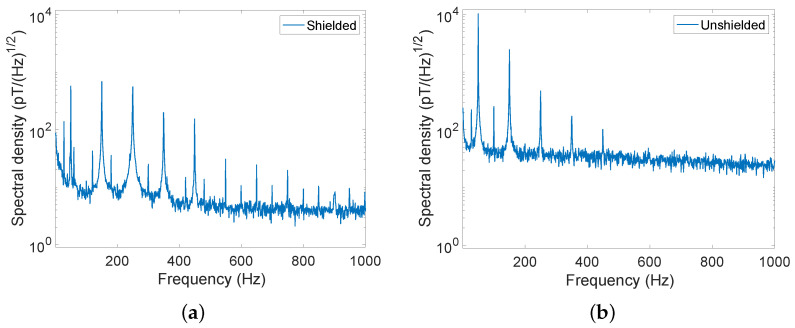
Fourier transforms of one-second time traces taken using a Bartington MAG690 magnetometer in (**a**) shielded and (**b**) unshielded conditions.

## Data Availability

Further data can be available from the authors upon request.

## Appendix A. Mesh Refinement Study

In all simulations performed in this paper, the domain was meshed with free tetrahedral elements consisting of 188,265 domain elements. We performed subsequent mesh refinements until no appreciable changes occurred in the simulated curves. Figure A1 represents the simulated magnetic field ratio ($|B_{ec}\left|/\right|B_{1}|$) for solid aluminium cylinders with different mesh sizes for domain elements: normal = 11,394; fine = 21,634; finer = 55,144; and extra-fine = 188,265. This is compared to the experimental data in Figure 6c. It can be seen that finer meshes result in the COMSOL simulation converging more closely to the experimental results.

## Appendix B. Allan Deviation and Sensitivity

In order to study the stability and sensitivity of the active detection setup, data were collected for 10 min without any object at a number of different frequencies (10 Hz, 120 Hz, 500 Hz and 1 kHz). The data were obtained with a Bartington MAG690 fluxgate magnetometer connected to an FPGA, which performed lock-in detection. This setup is identical to that used to collect data in Section 4. The Allan deviation (a measure of frequency stability) of these noise measurements can then be calculated. The measurements performed to calculate the Allan deviation were all taken in unshielded conditions. Figure A2 shows the Allan deviation of the in-phase component of these noise signals. The Allan deviation of the out-of-phase component is found to be similar to that of the in-phase component. In these figures, it can be seen that having both coils on does not make the signal more unstable than having only one of them on. In the active detection setup, both coils are used to cancel the background fields in the lab. When both coils are on, the smallest detectable fields (the smallest Allan deviation) are 51 pT at 10 Hz, 35 pT at 120 Hz, 62 pT at 500 Hz, and 70 pT at 1 kHz, which occur at gate times τ∼1–10 s. In Figure A2, the noise of the fluxgate with both coils off can be understood, as the Allan deviation decreases across all gate times. When the other coils are turned on, either individually or together, the Allan deviation initially decreases and then increases again after a certain amount of time. This is most likely due to drifts in the current through the coils. In order to improve the stability of the coils, a less noisy current supply could be used.

In order to find the sensitivity of the fluxgate magnetometer, one second of data was collected from the magnetometer, then the power spectral density was calculated from the time trace. This was carried out both inside a magnetic shield with all end caps on (shielded conditions), as shown in Figure A3a, and in unshielded conditions, as shown in Figure A3b. By taking these measurements in shielded conditions, the intrinsic sensitivity of the fluxgate could be determined. From Figure A3, it can be seen that the environmental noise at all frequencies in unshielded conditions is about an order of magnitude larger than the intrinsic noise of the sensor. Hence, in unshielded conditions the setup is limited by the environmental noise in the lab. In Figure A3b, the sensitivity at 10 Hz, 120 Hz, 500 Hz, and 1000 Hz is found to be ∼25 pT/$\sqrt{\mathrm{Hz}}$, ∼30 pT/$\sqrt{\mathrm{Hz}}$, ∼40 pT/$\sqrt{\mathrm{Hz}}$, and ∼50 pT/$\sqrt{\mathrm{Hz}}$, respectively.

## Appendix C. Calculating Conductivity and Permeability

The conductivity of aluminium and steel, as well as the permeability of steel, can be determined using theory [21,23]. Figure 1 shows the setup with the corresponding theoretical parameters for a sample of radius *r* and thickness *t*. The distance from the centre of the excitation coil to the centre of the sample is denoted by *a*, while the distance from the centre of the sample to the detection point of the magnetometer is *a’*.

### Appendix C.1. Aluminium

The conductivity of the aluminium sample used in this study can be determined using the low-frequency limit of the data shown in Figure 6c. The equations in [23] are altered to match the setup used in Section 4.1. The current induced in the cylinder in a thin area from the radial distance ρ to ρ+dρ is provided by

(A1) $$dI=\frac{mt}{2\pi \delta^{2}}\frac{\rho }{{\left(a^{2}+\rho^{2}\right)}^{3/2}}d\rho ,$$

where *m* is the magnetic dipole moment of the coil and δ is the skin depth [23]. This current then induces a magnetic field measured at the detection point as

(A2) $$dB_{\mathrm{ec}}=\frac{\mu_{0}dI}{2}\frac{\rho^{2}}{{\left(\rho^{2}+a\prime {}^{2}\right)}^{3/2}}.$$

Integrating from the centre of the cylinder ρ=0 to the radius of the cylinder ρ=r provides the total field induced by the eddy currents at the detection point of the magnetometer. Thus, for a≠a′,

(A3) $$B_{\mathrm{ec}}=\frac{mt\mu_{0}}{4\pi \delta^{2}}\left(\frac{a^{2}\left(2a\prime {}^{2}+r^{2}\right)+a\prime {}^{2}r^{2}}{(a^{2}-a\prime {}^{2}{)}^{2}\sqrt{a^{2}+r^{2}}\sqrt{a\prime {}^{2}+r^{2}}}-\frac{2aa\prime }{(a^{2}-a\prime {}^{2}{)}^{2}}\right).$$

Note that if a=a′, then Equation (A3) is not defined. This is due to the integral simplifying to a simpler equation, which is shown in [23]. Both equations tend to the same limit, as a→a′. By substituting the skin depth $\delta^{2}=1/\left(f\pi \mu \sigma \right)$ and dividing through $fB_{1}=f\mu_{0}m/2{\left(a+a\prime \right)}^{3}\pi$ at the detection point of the magnetometer, it can be found that

(A4) $$\frac{B_{\mathrm{ec}}}{B_{1}f}=\frac{t\pi \mu \sigma }{2}\frac{(a+a\prime {)}^{3}}{(a^{2}-a\prime {}^{2}{)}^{2}}\left(\frac{a^{2}\left(2a\prime {}^{2}+r^{2}\right)+a\prime {}^{2}r^{2}}{\sqrt{a^{2}+r^{2}}\sqrt{a\prime {}^{2}+r^{2}}}-2aa\prime \right),$$

where the only unknown is σ. Here, the left-hand side is provided by the gradient of the magnetic field ratio at the low-frequency limit. Using a fit function in MATLAB and the experimental data for the aluminium cylinder in Figure 7, we obtain a conductivity of 25.5(±1.8) MS/m, which is in agreement with the available values for 6061 T6 aluminium.

### Appendix C.2. Steel

In order to calculate the conductivity and magnetic permeability of the steel sample used here, the equations in [21,24] must to be altered to match the setup in Figure 1. Using data from Section 4.1, the conductivity and permeability of our 440c steel cylinder can be determined.

The primary magnetic field at the centre of the cylinder is provided by

(A5) $$B_{1}\left(z=a\right)=\frac{\mu_{0}m}{2\pi a^{3}},$$

where *m* is the magnetic moment of the excitation coil, *a* is the distance from the centre of the excitation coil to the centre of the object, and $\mu_{0}$ is the vacuum permeability. This induces a secondary field at the position of the sensor equal to

(A6) $$B_{\mathrm{ec}}\left(z=a+a\prime \right)=\frac{\mu_{0}m_{\mathrm{ec}}}{2\pi a\prime {}^{3}},$$

where *a* is the distance from the centre of the object to the detection point of the fluxgate magnetometer and $m_{\mathrm{ec}}$ is the induced magnetic moment in the object. For a sphere of radius *r*, the magnetic moment is provided by

(A7) $$m_{\mathrm{ec}}=\frac{2\pi r^{3}B_{1}\left(z=a\right)}{\mu_{0}}\frac{2\left(\mu_{r}-1\right)j_{0}\left(kr\right)+\left(2\mu_{r}+1\right)j_{2}\left(kr\right)}{\left(\mu_{r}+2\right)j_{0}\left(kr\right)+\left(\mu_{r}-1\right)j_{2}\left(kr\right)},$$

where $j_{0}$ and $j_{2}$ are spherical Bessel functions and $\mu_{r}$ is the relative permeability to be determined [21]. The propagation constant is provided by $k=\sqrt{\mu \varepsilon \omega^{2}+i\mu \sigma \omega }$, where ε is the permittivity of the sample, $\mu =\mu_{0}\mu_{r}$, and ω=2πν.

By combining Equations (A5) and (A6) at the detection point of the magnetometer, the following equation for the magnetic field ratio can be obtained:

(A8) $$\frac{B_{\mathrm{ec}}\left(z=a+a\prime \right)}{B_{1}\left(z=a+a\prime \right)}=\frac{r^{3}{\left(a+a\prime \right)}^{3}}{{\left(aa\prime \right)}^{3}}\frac{2\left(\mu_{r}-1\right)j_{0}\left(kr\right)+\left(2\mu_{r}+1\right)j_{2}\left(kr\right)}{\left(\mu_{r}+1\right)j_{0}\left(kr\right)+\left(\mu_{r}-1\right)j_{2}\left(kr\right)}.$$

This equation assumes a uniform RF magnetic field [21] across a sphere. However, the data we collected were for a cylinder with a 2 cm radius. As the cylinder has a finite radius, the primary field across it is not completely uniform. Nonetheless, we found that our experimental results are well described by Equation (A8) if it is multiplied by a scale factor. Using a fit function in MATLAB and the parameters outlined in Section 2, we can determine $\mu_{r}$, σ and the scale factor for 440c steel. The scale factor was calculated to be 0.56(±0.01), meaning that the theoretical approach overestimated $|B_{\mathrm{ec}}\left|/\right|B_{1}|$ by 79 (±3)%. The permeability and conductivity values were calculated to be $\mu_{r}=50\;\left(\pm 15\right)$ and σ=1.67(±0.20) MS/m. The function was fitted to the real and imaginary components of the field ratio $|B_{\mathrm{ec}}\left|/\right|B_{1}|$. This method can be used to calculate the conductivity of aluminium as well, as it is known that $\mu_{r}=1$. Figure A4 shows a comparison between the theoretical, simulated, and experimental data with and without the scale factor.
